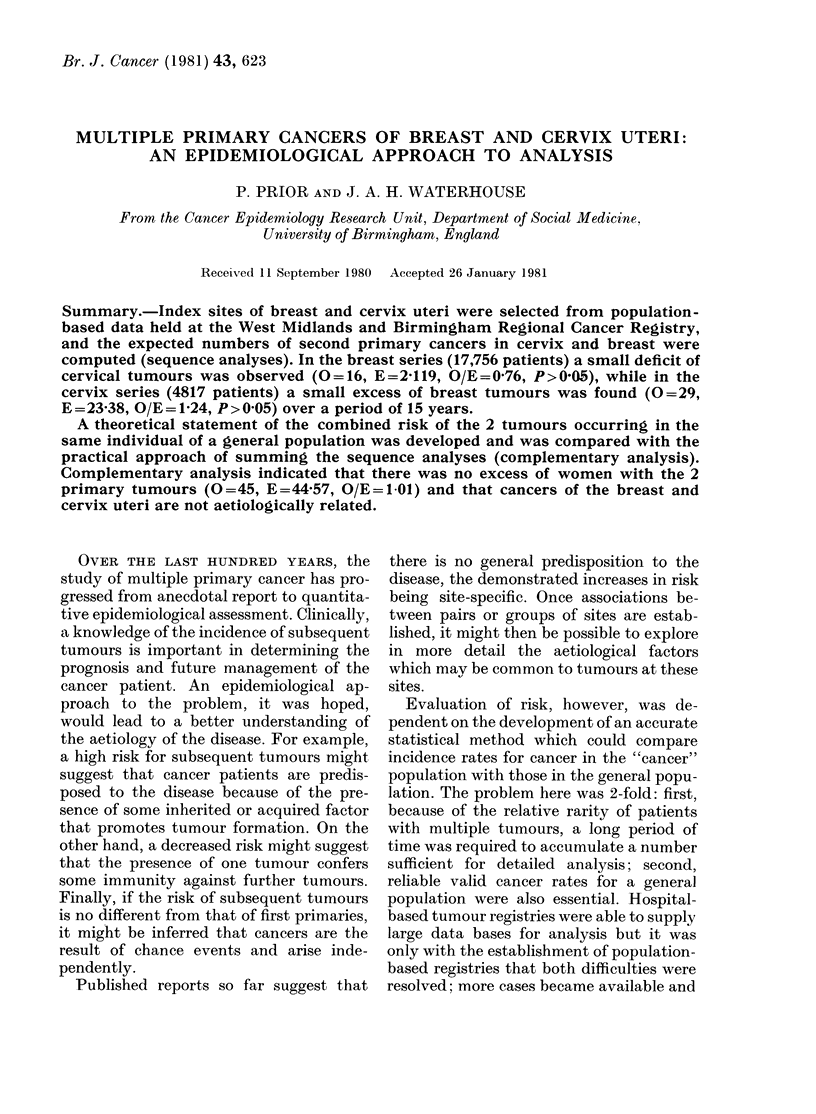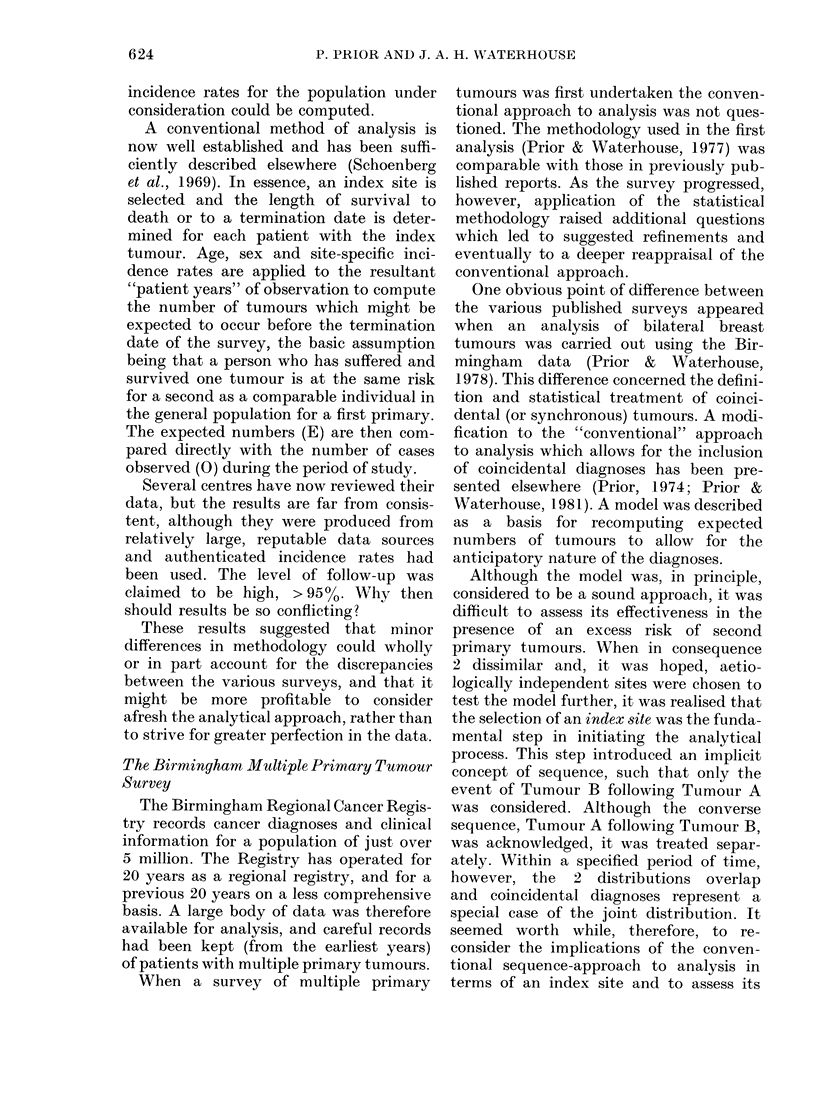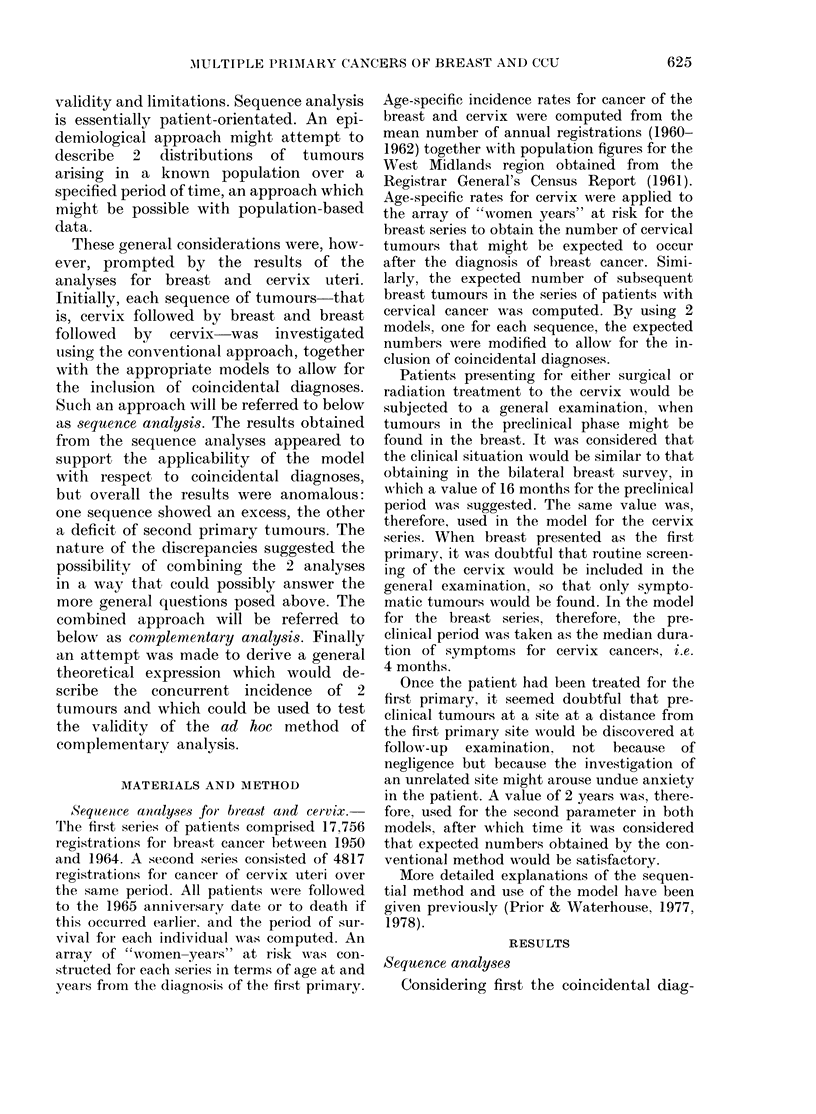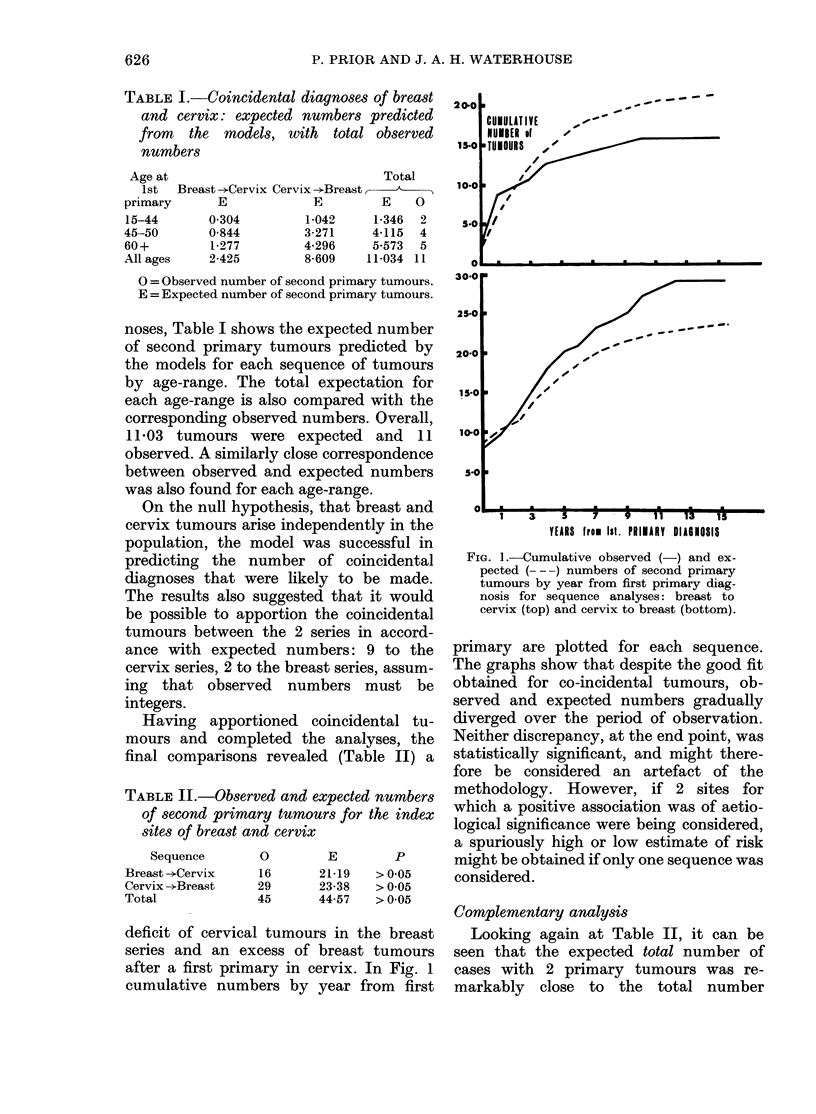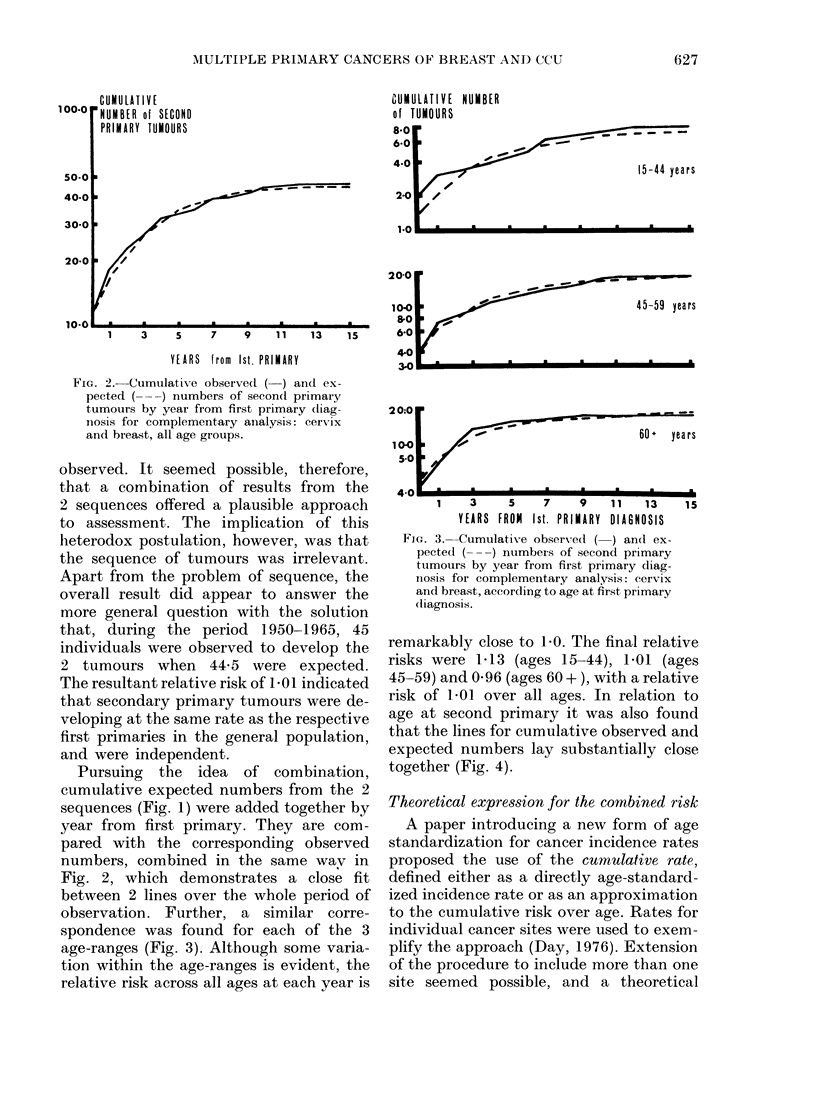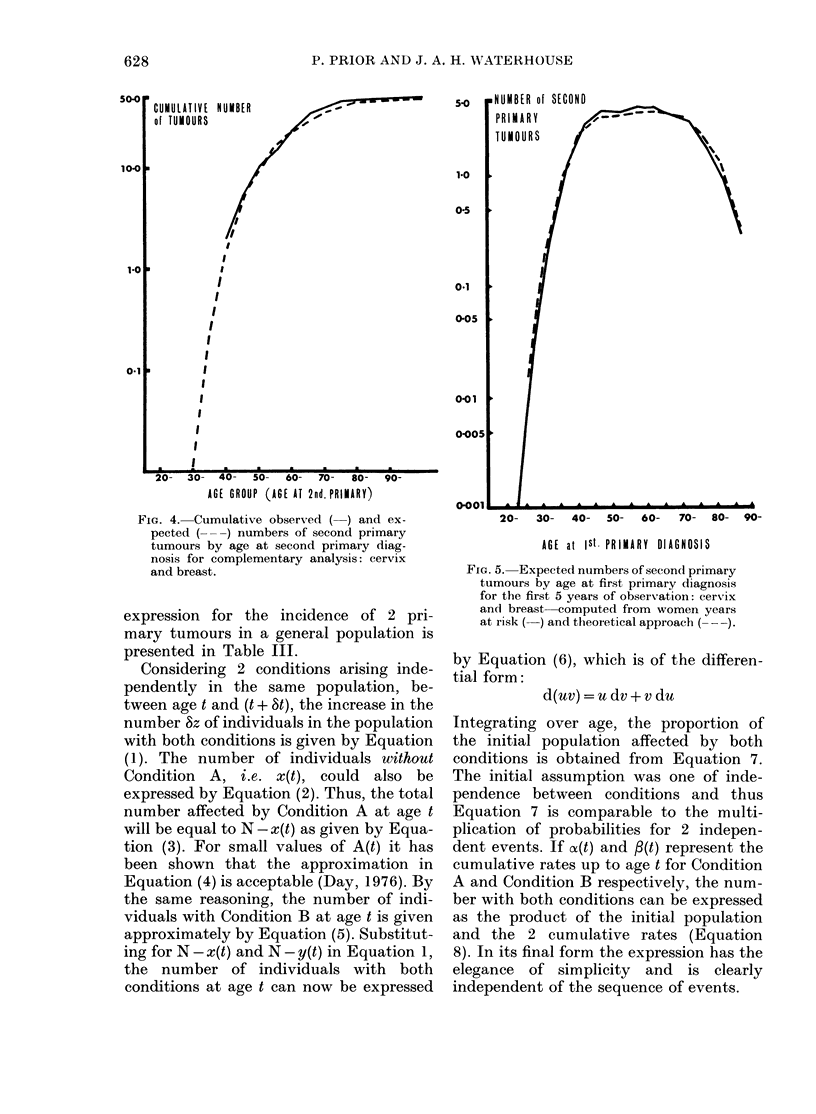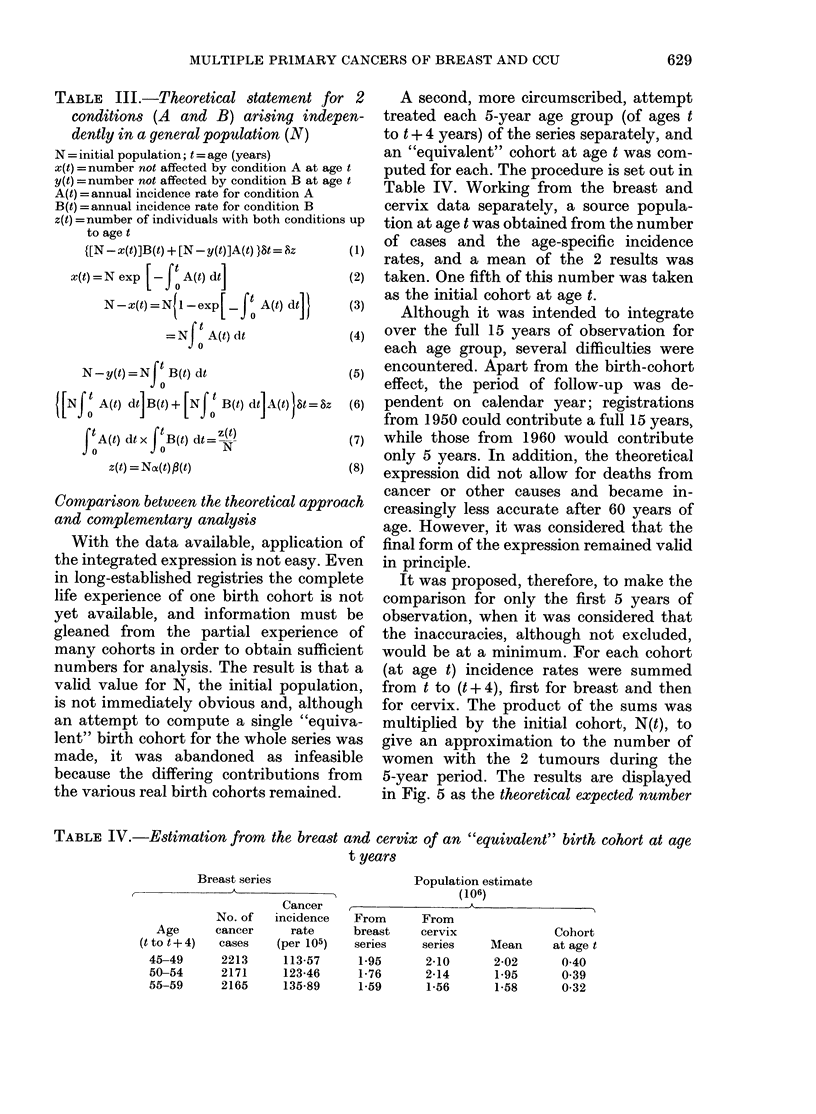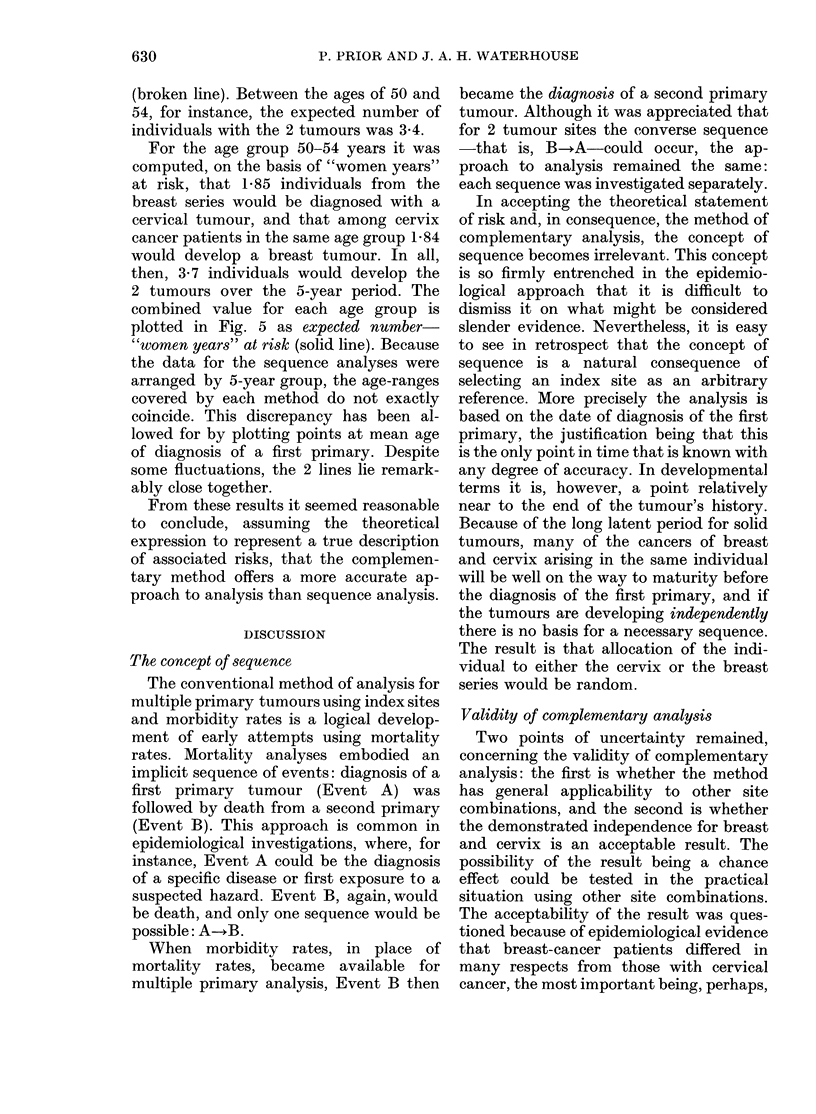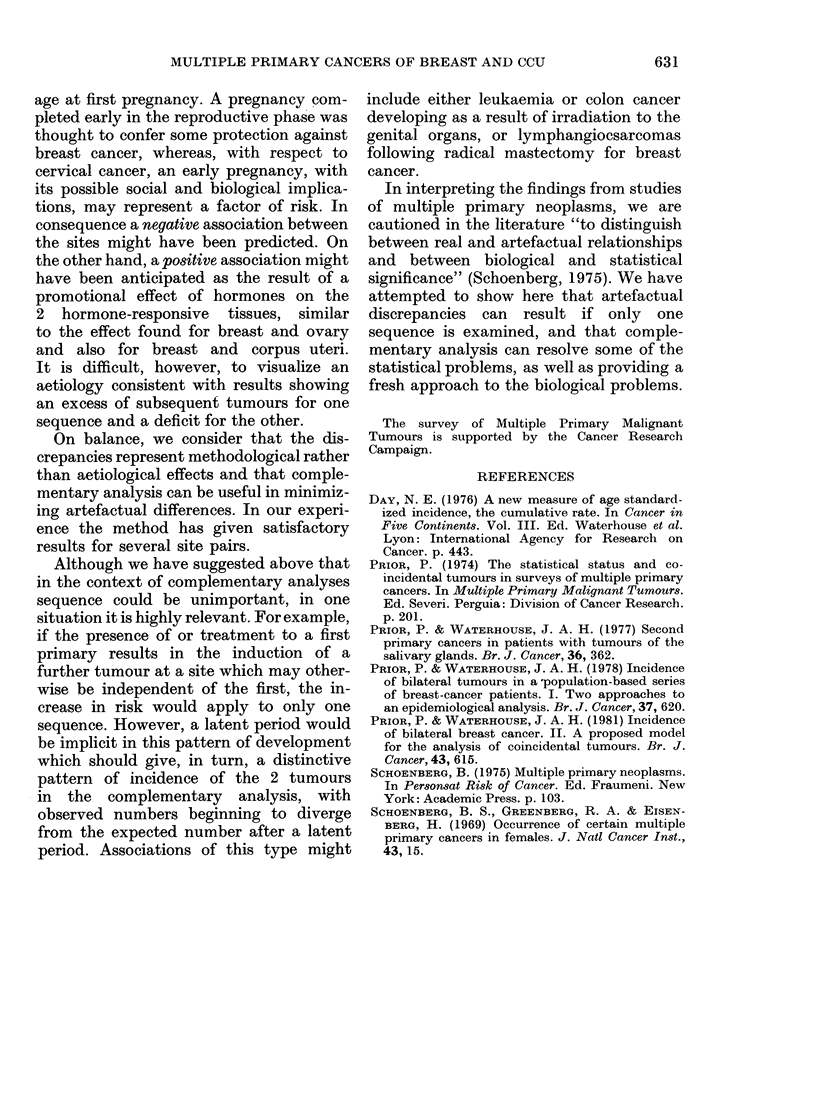# Multiple primary cancers of breast and cervix uteri: An epidemiological approach to analysis

**DOI:** 10.1038/bjc.1981.92

**Published:** 1981-05

**Authors:** P. Prior, J. A. H. Waterhouse

## Abstract

Index sites of breast and cervix uteri were selected from populationbased data held at the West Midlands and Birmingham Regional Cancer Registry, and the expected numbers of second primary cancers in cervix and breast were computed (sequence analyses). In the breast series (17,756 patients) a small deficit of cervical tumours was observed (O = 16, E = 2·119, O/E = 0·76, *P* > 0·05), while in the cervix series (4817 patients) a small excess of breast tumours was found (O = 29, E = 23·38, O/E = 1·24, *P* > 0·05) over a period of 15 years.

A theoretical statement of the combined risk of the 2 tumours occurring in the same individual of a general population was developed and was compared with the practical approach of summing the sequence analyses (complementary analysis). Complementary analysis indicated that there was no excess of women with the 2 primary tumours (O = 45, E = 44·57, O/E = 1·01) and that cancers of the breast and cervix uteri are not aetiologically related.


*Br. J. Cancer* (1981) **43**, 623

### MULTIPLE PRIMARY CANCERS OF BREAST AND CERVIX UTERI: AN EPIDEMIOLOGICAL APPROACH TO ANALYSIS

P. PRIOR AND J. A. H. WATERHOUSE

*From the Cancer Epidemiology Research Unit, Department of Social Medicine, University of Birmingham, England*

Received 11 September 1980 Accepted 26 January 1981

**Summary.—Index sites of breast and cervix uteri were selected from population-based data held at the West Midlands and Birmingham Regional Cancer Registry, and the expected numbers of second primary cancers in cervix and breast were computed (sequence analyses). In the breast series (17,756 patients) a small deficit of cervical tumours was observed (O=16, E=2·119, O/E=0·76, $P>0·05$), while in the cervix series (4817 patients) a small excess of breast tumours was found (O=29, E=23·38, O/E=1·24, $P>0·05$) over a period of 15 years.**

**A theoretical statement of the combined risk of the 2 tumours occurring in the same individual of a general population was developed and was compared with the practical approach of summing the sequence analyses (complementary analysis). Complementary analysis indicated that there was no excess of women with the 2 primary tumours (O=45, E=44·57, O/E=1·01) and that cancers of the breast and cervix uteri are not aetiologically related.**

OVER THE LAST HUNDRED YEARS, the study of multiple primary cancer has progressed from anecdotal report to quantitative epidemiological assessment. Clinically, a knowledge of the incidence of subsequent tumours is important in determining the prognosis and future management of the cancer patient. An epidemiological approach to the problem, it was hoped, would lead to a better understanding of the aetiology of the disease. For example, a high risk for subsequent tumours might suggest that cancer patients are predisposed to the disease because of the presence of some inherited or acquired factor that promotes tumour formation. On the other hand, a decreased risk might suggest that the presence of one tumour confers some immunity against further tumours. Finally, if the risk of subsequent tumours is no different from that of first primaries, it might be inferred that cancers are the result of chance events and arise independently.

Published reports so far suggest that there is no general predisposition to the disease, the demonstrated increases in risk being site-specific. Once associations between pairs or groups of sites are established, it might then be possible to explore in more detail the aetiological factors which may be common to tumours at these sites.

Evaluation of risk, however, was dependent on the development of an accurate statistical method which could compare incidence rates for cancer in the "cancer" population with those in the general population. The problem here was 2-fold: first, because of the relative rarity of patients with multiple tumours, a long period of time was required to accumulate a number sufficient for detailed analysis; second, reliable valid cancer rates for a general population were also essential. Hospital-based tumour registries were able to supply large data bases for analysis but it was only with the establishment of population-based registries that both difficulties were resolved; more cases became available and incidence rates for the population under consideration could be computed.

A conventional method of analysis is now well established and has been sufficiently described elsewhere (Schoenberg *et al.*, 1969). In essence, an index site is selected and the length of survival to death or to a termination date is determined for each patient with the index tumour. Age, sex and site-specific incidence rates are applied to the resultant "patient years" of observation to compute the number of tumours which might be expected to occur before the termination date of the survey, the basic assumption being that a person who has suffered and survived one tumour is at the same risk for a second as a comparable individual in the general population for a first primary. The expected numbers (E) are then compared directly with the number of cases observed (O) during the period of study.

Several centres have now reviewed their data, but the results are far from consistent, although they were produced from relatively large, reputable data sources and authenticated incidence rates had been used. The level of follow-up was claimed to be high, $>95\%$. Why then should results be so conflicting?

These results suggested that minor differences in methodology could wholly or in part account for the discrepancies between the various surveys, and that it might be more profitable to consider afresh the analytical approach, rather than to strive for greater perfection in the data.

*The Birmingham Multiple Primary Tumour Survey*

The Birmingham Regional Cancer Registry records cancer diagnoses and clinical information for a population of just over 5 million. The Registry has operated for 20 years as a regional registry, and for a previous 20 years on a less comprehensive basis. A large body of data was therefore available for analysis, and careful records had been kept (from the earliest years) of patients with multiple primary tumours.

When a survey of multiple primary tumours was first undertaken the conventional approach to analysis was not questioned. The methodology used in the first analysis (Prior & Waterhouse, 1977) was comparable with those in previously published reports. As the survey progressed, however, application of the statistical methodology raised additional questions which led to suggested refinements and eventually to a deeper reappraisal of the conventional approach.

One obvious point of difference between the various published surveys appeared when an analysis of bilateral breast tumours was carried out using the Birmingham data (Prior & Waterhouse, 1978). This difference concerned the definition and statistical treatment of coincidental (or synchronous) tumours. A modification to the "conventional" approach to analysis which allows for the inclusion of coincidental diagnoses has been presented elsewhere (Prior, 1974; Prior & Waterhouse, 1981). A model was described as a basis for recomputing expected numbers of tumours to allow for the anticipatory nature of the diagnoses.

Although the model was, in principle, considered to be a sound approach, it was difficult to assess its effectiveness in the presence of an excess risk of second primary tumours. When in consequence 2 dissimilar and, it was hoped, aetiologically independent sites were chosen to test the model further, it was realised that the selection of an *index site* was the fundamental step in initiating the analytical process. This step introduced an implicit concept of sequence, such that only the event of Tumour B following Tumour A was considered. Although the converse sequence, Tumour A following Tumour B, was acknowledged, it was treated separately. Within a specified period of time, however, the 2 distributions overlap and coincidental diagnoses represent a special case of the joint distribution. It seemed worth while, therefore, to reconsider the implications of the conventional sequence-approach to analysis in terms of an index site and to assess its validity and limitations. Sequence analysis is essentially patient-orientated. An epidemiological approach might attempt to describe 2 distributions of tumours arising in a known population over a specified period of time, an approach which might be possible with population-based data.

These general considerations were, however, prompted by the results of the analyses for breast and cervix uteri. Initially, each sequence of tumours—that is, cervix followed by breast and breast followed by cervix—was investigated using the conventional approach, together with the appropriate models to allow for the inclusion of coincidental diagnoses. Such an approach will be referred to below as *sequence analysis*. The results obtained from the sequence analyses appeared to support the applicability of the model with respect to coincidental diagnoses, but overall the results were anomalous: one sequence showed an excess, the other a deficit of second primary tumours. The nature of the discrepancies suggested the possibility of combining the 2 analyses in a way that could possibly answer the more general questions posed above. The combined approach will be referred to below as *complementary analysis*. Finally an attempt was made to derive a general theoretical expression which would describe the concurrent incidence of 2 tumours and which could be used to test the validity of the *ad hoc* method of complementary analysis.

### MATERIALS AND METHOD

*Sequence analyses for breast and cervix.*—The first series of patients comprised 17,756 registrations for breast cancer between 1950 and 1964. A second series consisted of 4817 registrations for cancer of cervix uteri over the same period. All patients were followed to the 1965 anniversary date or to death if this occurred earlier, and the period of survival for each individual was computed. An array of "women–years" at risk was constructed for each series in terms of age at and years from the diagnosis of the first primary. Age-specific incidence rates for cancer of the breast and cervix were computed from the mean number of annual registrations (1960–1962) together with population figures for the West Midlands region obtained from the Registrar General's Census Report (1961). Age-specific rates for cervix were applied to the array of "women years" at risk for the breast series to obtain the number of cervical tumours that might be expected to occur after the diagnosis of breast cancer. Similarly, the expected number of subsequent breast tumours in the series of patients with cervical cancer was computed. By using 2 models, one for each sequence, the expected numbers were modified to allow for the inclusion of coincidental diagnoses.

Patients presenting for either surgical or radiation treatment to the cervix would be subjected to a general examination, when tumours in the preclinical phase might be found in the breast. It was considered that the clinical situation would be similar to that obtaining in the bilateral breast survey, in which a value of 16 months for the preclinical period was suggested. The same value was, therefore, used in the model for the cervix series. When breast presented as the first primary, it was doubtful that routine screening of the cervix would be included in the general examination, so that only symptomatic tumours would be found. In the model for the breast series, therefore, the preclinical period was taken as the median duration of symptoms for cervix cancers, *i.e.* 4 months.

Once the patient had been treated for the first primary, it seemed doubtful that preclinical tumours at a site at a distance from the first primary site would be discovered at follow-up examination, not because of negligence but because the investigation of an unrelated site might arouse undue anxiety in the patient. A value of 2 years was, therefore, used for the second parameter in both models, after which time it was considered that expected numbers obtained by the conventional method would be satisfactory.

More detailed explanations of the sequential method and use of the model have been given previously (Prior & Waterhouse, 1977, 1978).

### RESULTS

### *Sequence analyses*

Considering first the coincidental diag-

TABLE I.—*Coincidental diagnoses of breast and cervix: expected numbers predicted from the models, with total observed numbers*

| Age at 1st primary | Breast→Cervix E | Cervix→Breast E | Total E | Total O |
|---|---|---|---|---|
| 15–44 | 0·304 | 1·042 | 1·346 | 2 |
| 45–50 | 0·844 | 3·271 | 4·115 | 4 |
| 60+ | 1·277 | 4·296 | 5·573 | 5 |
| All ages | 2·425 | 8·609 | 11·034 | 11 |

O = Observed number of second primary tumours.
E = Expected number of second primary tumours.

noses, Table I shows the expected number of second primary tumours predicted by the models for each sequence of tumours by age-range. The total expectation for each age-range is also compared with the corresponding observed numbers. Overall, 11·03 tumours were expected and 11 observed. A similarly close correspondence between observed and expected numbers was also found for each age-range.

On the null hypothesis, that breast and cervix tumours arise independently in the population, the model was successful in predicting the number of coincidental diagnoses that were likely to be made. The results also suggested that it would be possible to apportion the coincidental tumours between the 2 series in accordance with expected numbers: 9 to the cervix series, 2 to the breast series, assuming that observed numbers must be integers.

Having apportioned coincidental tumours and completed the analyses, the final comparisons revealed (Table II) a

TABLE II.—*Observed and expected numbers of second primary tumours for the index sites of breast and cervix*

| Sequence | O | E | *P* |
|---|---|---|---|
| Breast→Cervix | 16 | 21·19 | > 0·05 |
| Cervix→Breast | 29 | 23·38 | > 0·05 |
| Total | 45 | 44·57 | > 0·05 |

deficit of cervical tumours in the breast series and an excess of breast tumours after a first primary in cervix. In Fig. 1 cumulative numbers by year from first

FIG. 1.—Cumulative observed (—) and expected (- - -) numbers of second primary tumours by year from first primary diagnosis for sequence analyses: breast to cervix (top) and cervix to breast (bottom).

primary are plotted for each sequence. The graphs show that despite the good fit obtained for co-incidental tumours, observed and expected numbers gradually diverged over the period of observation. Neither discrepancy, at the end point, was statistically significant, and might therefore be considered an artefact of the methodology. However, if 2 sites for which a positive association was of aetiological significance were being considered, a spuriously high or low estimate of risk might be obtained if only one sequence was considered.

*Complementary analysis*

Looking again at Table II, it can be seen that the expected *total* number of cases with 2 primary tumours was remarkably close to the total number

FIG. 2.—Cumulative observed (—) and expected (– – –) numbers of second primary tumours by year from first primary diagnosis for complementary analysis: cervix and breast, all age groups.

FIG. 3.—Cumulative observed (—) and expected (– – –) numbers of second primary tumours by year from first primary diagnosis for complementary analysis: cervix and breast, according to age at first primary diagnosis.

observed. It seemed possible, therefore, that a combination of results from the 2 sequences offered a plausible approach to assessment. The implication of this heterodox postulation, however, was that the sequence of tumours was irrelevant. Apart from the problem of sequence, the overall result did appear to answer the more general question with the solution that, during the period 1950–1965, 45 individuals were observed to develop the 2 tumours when 44·5 were expected. The resultant relative risk of 1·01 indicated that secondary primary tumours were developing at the same rate as the respective first primaries in the general population, and were independent.

Pursuing the idea of combination, cumulative expected numbers from the 2 sequences (Fig. 1) were added together by year from first primary. They are compared with the corresponding observed numbers, combined in the same way in Fig. 2, which demonstrates a close fit between 2 lines over the whole period of observation. Further, a similar correspondence was found for each of the 3 age-ranges (Fig. 3). Although some variation within the age-ranges is evident, the relative risk across all ages at each year is remarkably close to 1·0. The final relative risks were 1·13 (ages 15–44), 1·01 (ages 45–59) and 0·96 (ages 60 + ), with a relative risk of 1·01 over all ages. In relation to age at second primary it was also found that the lines for cumulative observed and expected numbers lay substantially close together (Fig. 4).

*Theoretical expression for the combined risk*

A paper introducing a new form of age standardization for cancer incidence rates proposed the use of the *cumulative rate*, defined either as a directly age-standardized incidence rate or as an approximation to the cumulative risk over age. Rates for individual cancer sites were used to exemplify the approach (Day, 1976). Extension of the procedure to include more than one site seemed possible, and a theoretical

FIG. 4.—Cumulative observed (—) and expected (- - -) numbers of second primary tumours by age at second primary diagnosis for complementary analysis: cervix and breast.

FIG. 5.—Expected numbers of second primary tumours by age at first primary diagnosis for the first 5 years of observation: cervix and breast—computed from women years at risk (—) and theoretical approach (- - -).

expression for the incidence of 2 primary tumours in a general population is presented in Table III.

Considering 2 conditions arising independently in the same population, between age $t$ and $(t+\delta t)$, the increase in the number $\delta z$ of individuals in the population with both conditions is given by Equation (1). The number of individuals *without* Condition A, *i.e.* $x(t)$, could also be expressed by Equation (2). Thus, the total number affected by Condition A at age $t$ will be equal to $\mathrm{N}-x(t)$ as given by Equation (3). For small values of $\mathrm{A}(t)$ it has been shown that the approximation in Equation (4) is acceptable (Day, 1976). By the same reasoning, the number of individuals with Condition B at age $t$ is given approximately by Equation (5). Substituting for $\mathrm{N}-x(t)$ and $\mathrm{N}-y(t)$ in Equation 1, the number of individuals with both conditions at age $t$ can now be expressed by Equation (6), which is of the differential form:

$$\mathrm{d}(uv) = u\,\mathrm{d}v + v\,\mathrm{d}u$$

Integrating over age, the proportion of the initial population affected by both conditions is obtained from Equation 7. The initial assumption was one of independence between conditions and thus Equation 7 is comparable to the multiplication of probabilities for 2 independent events. If $\alpha(t)$ and $\beta(t)$ represent the cumulative rates up to age $t$ for Condition A and Condition B respectively, the number with both conditions can be expressed as the product of the initial population and the 2 cumulative rates (Equation 8). In its final form the expression has the elegance of simplicity and is clearly independent of the sequence of events.

TABLE III.—*Theoretical statement for 2 conditions (A and B) arising independently in a general population (N)*

N = initial population; $t$ = age (years)
$x(t)$ = number *not* affected by condition A at age $t$
$y(t)$ = number *not* affected by condition B at age $t$
$A(t)$ = annual incidence rate for condition A
$B(t)$ = annual incidence rate for condition B
$z(t)$ = number of individuals with both conditions up to age $t$

$$\{[N - x(t)]B(t) + [N - y(t)]A(t)\}\delta t = \delta z \quad (1)$$

$$x(t) = N \exp\left[-\int_0^t A(t)\,dt\right] \quad (2)$$

$$N - x(t) = N\left\{1 - \exp\left[-\int_0^t A(t)\,dt\right]\right\} \quad (3)$$

$$= N\int_0^t A(t)\,dt \quad (4)$$

$$N - y(t) = N\int_0^t B(t)\,dt \quad (5)$$

$$\left\{\left[N\int_0^t A(t)\,dt\right]B(t) + \left[N\int_0^t B(t)\,dt\right]A(t)\right\}\delta t = \delta z \quad (6)$$

$$\int_0^t A(t)\,dt \times \int_0^t B(t)\,dt = \frac{z(t)}{N} \quad (7)$$

$$z(t) = N\alpha(t)\beta(t) \quad (8)$$

*Comparison between the theoretical approach and complementary analysis*

With the data available, application of the integrated expression is not easy. Even in long-established registries the complete life experience of one birth cohort is not yet available, and information must be gleaned from the partial experience of many cohorts in order to obtain sufficient numbers for analysis. The result is that a valid value for N, the initial population, is not immediately obvious and, although an attempt to compute a single "equivalent" birth cohort for the whole series was made, it was abandoned as infeasible because the differing contributions from the various real birth cohorts remained.

A second, more circumscribed, attempt treated each 5-year age group (of ages $t$ to $t+4$ years) of the series separately, and an "equivalent" cohort at age $t$ was computed for each. The procedure is set out in Table IV. Working from the breast and cervix data separately, a source population at age $t$ was obtained from the number of cases and the age-specific incidence rates, and a mean of the 2 results was taken. One fifth of this number was taken as the initial cohort at age $t$.

Although it was intended to integrate over the full 15 years of observation for each age group, several difficulties were encountered. Apart from the birth-cohort effect, the period of follow-up was dependent on calendar year; registrations from 1950 could contribute a full 15 years, while those from 1960 would contribute only 5 years. In addition, the theoretical expression did not allow for deaths from cancer or other causes and became increasingly less accurate after 60 years of age. However, it was considered that the final form of the expression remained valid in principle.

It was proposed, therefore, to make the comparison for only the first 5 years of observation, when it was considered that the inaccuracies, although not excluded, would be at a minimum. For each cohort (at age $t$) incidence rates were summed from $t$ to $(t+4)$, first for breast and then for cervix. The product of the sums was multiplied by the initial cohort, $N(t)$, to give an approximation to the number of women with the 2 tumours during the 5-year period. The results are displayed in Fig. 5 as the *theoretical expected number*

TABLE IV.—*Estimation from the breast and cervix of an "equivalent" birth cohort at age* t *years*

| | Breast series | | Population estimate ($10^6$) | | | |
|---|---|---|---|---|---|---|
| Age ($t$ to $t+4$) | No. of cancer cases | Cancer incidence rate (per $10^5$) | From breast series | From cervix series | Mean | Cohort at age $t$ |
| 45–49 | 2213 | 113·57 | 1·95 | 2·10 | 2·02 | 0·40 |
| 50–54 | 2171 | 123·46 | 1·76 | 2·14 | 1·95 | 0·39 |
| 55–59 | 2165 | 135·89 | 1·59 | 1·56 | 1·58 | 0·32 |

(broken line). Between the ages of 50 and 54, for instance, the expected number of individuals with the 2 tumours was 3·4.

For the age group 50–54 years it was computed, on the basis of "women years" at risk, that 1·85 individuals from the breast series would be diagnosed with a cervical tumour, and that among cervix cancer patients in the same age group 1·84 would develop a breast tumour. In all, then, 3·7 individuals would develop the 2 tumours over the 5-year period. The combined value for each age group is plotted in Fig. 5 as *expected number—"women years" at risk* (solid line). Because the data for the sequence analyses were arranged by 5-year group, the age-ranges covered by each method do not exactly coincide. This discrepancy has been allowed for by plotting points at mean age of diagnosis of a first primary. Despite some fluctuations, the 2 lines lie remarkably close together.

From these results it seemed reasonable to conclude, assuming the theoretical expression to represent a true description of associated risks, that the complementary method offers a more accurate approach to analysis than sequence analysis.

### DISCUSSION

### *The concept of sequence*

The conventional method of analysis for multiple primary tumours using index sites and morbidity rates is a logical development of early attempts using mortality rates. Mortality analyses embodied an implicit sequence of events: diagnosis of a first primary tumour (Event A) was followed by death from a second primary (Event B). This approach is common in epidemiological investigations, where, for instance, Event A could be the diagnosis of a specific disease or first exposure to a suspected hazard. Event B, again, would be death, and only one sequence would be possible: A→B.

When morbidity rates, in place of mortality rates, became available for multiple primary analysis, Event B then became the *diagnosis* of a second primary tumour. Although it was appreciated that for 2 tumour sites the converse sequence —that is, B→A—could occur, the approach to analysis remained the same: each sequence was investigated separately.

In accepting the theoretical statement of risk and, in consequence, the method of complementary analysis, the concept of sequence becomes irrelevant. This concept is so firmly entrenched in the epidemiological approach that it is difficult to dismiss it on what might be considered slender evidence. Nevertheless, it is easy to see in retrospect that the concept of sequence is a natural consequence of selecting an index site as an arbitrary reference. More precisely the analysis is based on the date of diagnosis of the first primary, the justification being that this is the only point in time that is known with any degree of accuracy. In developmental terms it is, however, a point relatively near to the end of the tumour's history. Because of the long latent period for solid tumours, many of the cancers of breast and cervix arising in the same individual will be well on the way to maturity before the diagnosis of the first primary, and if the tumours are developing *independently* there is no basis for a necessary sequence. The result is that allocation of the individual to either the cervix or the breast series would be random.

### *Validity of complementary analysis*

Two points of uncertainty remained, concerning the validity of complementary analysis: the first is whether the method has general applicability to other site combinations, and the second is whether the demonstrated independence for breast and cervix is an acceptable result. The possibility of the result being a chance effect could be tested in the practical situation using other site combinations. The acceptability of the result was questioned because of epidemiological evidence that breast-cancer patients differed in many respects from those with cervical cancer, the most important being, perhaps, age at first pregnancy. A pregnancy completed early in the reproductive phase was thought to confer some protection against breast cancer, whereas, with respect to cervical cancer, an early pregnancy, with its possible social and biological implications, may represent a factor of risk. In consequence a *negative* association between the sites might have been predicted. On the other hand, a *positive* association might have been anticipated as the result of a promotional effect of hormones on the 2 hormone-responsive tissues, similar to the effect found for breast and ovary and also for breast and corpus uteri. It is difficult, however, to visualize an aetiology consistent with results showing an excess of subsequent tumours for one sequence and a deficit for the other.

On balance, we consider that the discrepancies represent methodological rather than aetiological effects and that complementary analysis can be useful in minimizing artefactual differences. In our experience the method has given satisfactory results for several site pairs.

Although we have suggested above that in the context of complementary analyses sequence could be unimportant, in one situation it is highly relevant. For example, if the presence of or treatment to a first primary results in the induction of a further tumour at a site which may otherwise be independent of the first, the increase in risk would apply to only one sequence. However, a latent period would be implicit in this pattern of development which should give, in turn, a distinctive pattern of incidence of the 2 tumours in the complementary analysis, with observed numbers beginning to diverge from the expected number after a latent period. Associations of this type might include either leukaemia or colon cancer developing as a result of irradiation to the genital organs, or lymphangiocsarcomas following radical mastectomy for breast cancer.

In interpreting the findings from studies of multiple primary neoplasms, we are cautioned in the literature "to distinguish between real and artefactual relationships and between biological and statistical significance" (Schoenberg, 1975). We have attempted to show here that artefactual discrepancies can result if only one sequence is examined, and that complementary analysis can resolve some of the statistical problems, as well as providing a fresh approach to the biological problems.

The survey of Multiple Primary Malignant Tumours is supported by the Cancer Research Campaign.

### REFERENCES

Day, N. E. (1976) A new measure of age standardized incidence, the cumulative rate. In *Cancer in Five Continents*. Vol. III. Ed. Waterhouse *et al.* Lyon: International Agency for Research on Cancer. p. 443.

Prior, P. (1974) The statistical status and coincidental tumours in surveys of multiple primary cancers. In *Multiple Primary Malignant Tumours*. Ed. Severi. Perguia: Division of Cancer Research. p. 201.

Prior, P. & Waterhouse, J. A. H. (1977) Second primary cancers in patients with tumours of the salivary glands. *Br. J. Cancer*, **36**, 362.

Prior, P. & Waterhouse, J. A. H. (1978) Incidence of bilateral tumours in a population-based series of breast-cancer patients. I. Two approaches to an epidemiological analysis. *Br. J. Cancer*, **37**, 620.

Prior, P. & Waterhouse, J. A. H. (1981) Incidence of bilateral breast cancer. II. A proposed model for the analysis of coincidental tumours. *Br. J. Cancer*, **43**, 615.

Schoenberg, B. (1975) Multiple primary neoplasms. In *Personsat Risk of Cancer*. Ed. Fraumeni. New York: Academic Press. p. 103.

Schoenberg, B. S., Greenberg, R. A. & Eisenberg, H. (1969) Occurrence of certain multiple primary cancers in females. *J. Natl Cancer Inst.*, **43**, 15.